# Higher Breast Cancer Risk Among Immigrant Asian American Women Than Among US-Born Asian American Women

**DOI:** 10.5888/pcd16.180221

**Published:** 2019-02-14

**Authors:** Brittany N. Morey, Gilbert C. Gee, Ondine S. von Ehrenstein, Salma Shariff-Marco, Alison J. Canchola, Juan Yang, Laura Allen, Sandra S-J. Lee, Roxanna Bautista, Trish La Chica, Winston Tseng, Pancho Chang, Scarlett Lin Gomez

**Affiliations:** 1 University of California–Riverside, School of Public Policy, Riverside, California; 2 University of California–Los Angeles, Fielding School of Public Health, Department of Community Health Sciences, Los Angeles, California; 3 University of California, Los Angeles, Fielding School of Public Health, Department of Epidemiology, Los Angeles, California; 4 Cancer Prevention Institute of California, Fremont, California; 5 University of California, San Francisco, School of Medicine, Department of Epidemiology & Biostatistics, San Francisco, California; 6 University of California, San Francisco, Helen Diller Family Comprehensive Cancer Center, San Francisco, California; 7 Stanford University School of Medicine, Center for Biomedical Ethics, Stanford, California; 8 Rise Up Solutions, San Francisco, California; 9 Hawai‘i Public Health Institute, Honolulu, Hawai‘i; 10 University of California, Berkeley, Health Research for Action, Berkeley, California; 11 Ravenswood Family Health Center, East Palo Alto, California

## Abstract

**Introduction:**

Given rising rates of breast cancer in parts of Asia, immigrant Asian American women in the United States may have higher rates of breast cancer than previously anticipated. This study examined breast cancer risk among Asian American women by nativity and percentage of life lived in the United States, accounting for established breast cancer risk factors.

**Methods:**

We analyzed a breast cancer case-control data set of Asian American women living in the San Francisco Bay Area; this data set included 132 cases of women with breast cancer selected from a Surveillance, Epidemiology, and End Results cancer registry and 438 Asian American women without diagnosed breast cancer matched to cases by age and country of origin. We used logistic regression to compare 3 Asian American groups: US-born, immigrants who lived 50% or more of their life in the United States, and immigrants who lived less than 50% of their life in the United States.

**Results:**

In the minimally adjusted and fully adjusted models, both groups of immigrant Asian American women had higher risk of breast cancer than US-born Asian American women. In the fully adjusted model, compared with US-born Asian American women, immigrant Asian American women who lived more than 50% of their life in United States were on average 3 times as likely (odds ratio = 3.00; 95% confidence interval, 1.56–5.75) and immigrants who lived less than 50% of their life in United States were on average 2.46 times as likely (odds ratio = 2.46; 95% confidence interval, 1.21–4.99) to have breast cancer. We found no difference in fully adjusted odds ratios of having breast cancer between the 2 immigrant groups.

**Conclusion:**

This study provides preliminary evidence that breast cancer risk among immigrant Asian American women may be higher than among their US-born counterparts.

**Summary**

**What is already known on this topic?**

Research in the United States has found that among Asian Americans, risk for female breast cancer was higher among US-born women than among women born outside the United States.

**What is added by this report?**

This report finds that this trend, in a more recent cohort of Asian Americans, may be shifting, such that breast cancer risk is higher among women who are immigrants compared with those who are US-born.

**What are the implications for public health practice?**

There may be an increased need for breast cancer treatment services for immigrant Asian Americans as well as for continued efforts to increase access to mammograms among all Asian American women.

## Introduction

Research in the United States has consistently found that for racial/ethnic minority populations such as Asian Americans, those not born in the United States have lower rates of female breast cancer than their US-born counterparts (1,2). However, this trend may be shifting among recent waves of Asian American immigrants.

Worldwide, breast cancer incidence is high in North America and relatively low in Asia (3). Although breast cancer rates in the United States have stabilized since the 2000s, rates are increasing rapidly in East and Southeast Asia, with the highest rates found in urban and affluent areas (3–6). These trends are possibly due to the effects of globalization and economic development on increased screening, lower parity, delayed childbirth, decreased breastfeeding, and sedentary lifestyles — all factors that increase breast cancer rates (3,7,8).

Current US immigration policies have led to the influx of highly skilled Asian immigrants who perhaps have a higher socioeconomic status than previous immigrant groups. In 2013, 51% of recent East and South Asian immigrants in the United States had at least a college degree; in 1970, only 20% of all immigrant arrivals had this level of education (9). High socioeconomic status is related to increased risk for breast cancer in numerous populations (10). Consistent with these observations, a recent analysis showed that breast cancer rates are increasing among most Asian American groups in California (11). Asian immigrants may arrive in the United States with higher risk for latent breast cancer than previous immigrant cohorts (12).

Our study adds to the existing literature by describing how breast cancer risk among Asian American women varies by nativity status and percentage of life lived in the United States, accounting for established breast cancer risk factors, and it is among the first to do so. We hypothesized that 1) breast cancer risk would differ by nativity, 2) a greater percentage of life lived in the United States would be associated with higher breast cancer risk, and 3) modifiable risk factors, including reproductive history and body mass index (BMI) (7,13), would attenuate these differences by nativity and percentage of life in the United States.

## Methods

We used a population-based case-control data set of Asian American women. We collected data from the Asian Community Health Initiative, a case-control study of breast cancer among Asian American women in the San Francisco Bay Area (14). The San Francisco Bay Area is an appropriate study location because it has the highest concentration of Asian Americans in the United States outside Hawai‘i, with 29% of the population (1.7 million) identifying as Asian American in the 2010 US census (15). Asian American women with breast cancer diagnosed during 2005–2009 were sampled from a population-based source — the Greater Bay Area Cancer Registry — part of the Surveillance, Epidemiology, and End Results (SEER) Program and the state-mandated California Cancer Registry. In a comparison of women with breast cancer in our sample with women in the California Cancer Registry, our sample was found to be representative of the source population.

Because selection bias can result from relying on a single recruitment method, the Asian Community Health Initiative used several methods to recruit women for the control sample (16). The Initiative used 5 strategies to recruit participants without breast cancer; these controls were used to represent the population of Asian American women at risk for breast cancer in the San Francisco Bay Area. The first strategy recruited participants from community health centers. The second strategy recruited participants by using email blasts through Army of Women, a volunteer registry of women with and without breast cancer who are interested in participating in breast cancer research (www.armyofwomen.org). The third strategy used monthly advertisements and posts on Craigslist, Facebook, Twitter, and listservs reaching Asian Americans. A fourth strategy used traditional address-based sampling of a randomly generated sample of 3,000 residential addresses of people with Asian American surnames; this strategy yielded a response rate of less than 2%. The fifth strategy involved disseminating flyers at health fairs, senior centers, community events, and fundraisers.

Initiative researchers frequency-matched controls to cases by Asian country of origin (Chinese, Filipina, and other Asian) and age (20–39, 40–59, and ≥60 y) in a 3:1 ratio of controls to cases. Researchers found the control sample to be representative of the overall population of Asian American women in the San Francisco Bay Area in comparisons of key demographic characteristics with data from the California Health Interview Survey (CHIS) (14).

Recruitment took place from March 2013 through October 2014 and yielded an analytical sample of 570 Asian American women consisting of 132 cases and 438 controls. Survey data were collected through telephone interviews and self-administered questionnaires in English, Chinese, or Tagalog. Written materials for Chinese and Tagalog were translated and independently back-translated. Participants received a $30 check for completing the telephone interview. Participants in the second-phase self-administered survey received an additional $15. Participants consisted of Chinese (53%), Filipina (20%), and other Asian American (27%) women aged 22 to 87 (mean age, 52). Among immigrant women, the average age at immigration was 22 (standard deviation, 19). All study procedures were approved by the ethical review boards at the Cancer Prevention Institute of California, University of California–Los Angeles, and the University of California–Riverside.

### Study variables

The outcome was breast cancer (1 = clinical diagnosis of breast cancer; 0 = no diagnosis). The independent variable of interest was nativity and percentage of life lived in the United States (US-born, immigrant with ≥50% of life lived in the United States; immigrant with <50% of life lived in the United States).

We adjusted for the following established breast cancer risk factors: pregnancy history (age at first birth <25 y, age at first birth 25–29 y, age at first birth 30–34 y, age at first birth ≥35 y, never had a pregnancy that lasted ≥7 months), family history of breast cancer (1 = mother, sister, or daughter had breast cancer; 0 = no immediate family member [mother, sister, or daughter] had breast cancer), and menopausal status and use of hormone replacement therapy (HRT) (premenopausal, postmenopausal and no history of using HRT, postmenopausal and history of using HRT). We also adjusted for BMI, calculated as the respondents’ reported weight in kilograms divided by the square of their height in meters. We adjusted for BMI because it is positively associated with higher risk of breast cancer among postmenopausal women (13) and because higher BMI is also commonly associated with greater acculturation among immigrant groups (17). We chose BMI cutoff points (<23, 24–26, or ≥27) that are based on research that found higher risk of chronic disease at lower BMIs among Asian populations than among the general population (18).

Additional covariates were socioeconomic status, operationalized as education level (college graduate, some college, high school diploma or less), home ownership (1 = homeowner; 0 = renter or non-homeowner), and health insurance status (1 = public insurance or not insured; 0 = private insurance). All study variables were self-reported.

### Statistical analysis

For each study variable, we calculated frequency and percentage by breast cancer status. We then used unconditional logistic regression to estimate odds ratios (ORs) and 95% confidence intervals (CIs) of breast cancer risk. Models were minimally adjusted for age group (20–39, 40–59, and ≥60) and country of origin (Chinese, Filipina, and other) (19). Fully adjusted models included measures of socioeconomic status and known breast cancer risk factors: education, home ownership, health insurance status, pregnancy history, family history of breast cancer, menopausal status and HRT use, and BMI. We conducted analyses by using Stata version 15 (StataCorp LLC). We conducted an additional sensitivity analysis, replacing percentage of life lived in the United States with other measures that used age at immigration (data available upon request). The results were similar when percentage of life lived in the United States was replaced with immigration before and after age 18. We also examined the association between breast cancer and immigration before or after menarche. However, too few women in the sample immigrated before menarche for these results to be conclusive.

Additionally, we analyzed whether other risk factors were associated with breast cancer, including physical activity, smoking status, alcohol use, and oral contraceptive use. In this sample none of these variables were associated with having breast cancer, and they were excluded from the final model.

## Results

A lower percentage of Asian American women with breast cancer (17% [22 of 132]) were US-born compared with controls (33% [144 of 438]) (Table). A greater percentage of Asian American women with breast cancer (42% [56 of 132]) were immigrants and lived more than 50% of their lives in the United States compared with controls (25% [110 of 438]). The percentage of home ownership was higher among Asian American women with breast cancer (77% [102 of 132]) than among controls (63% [276 of 438]). Compared with controls, Asian American women with breast cancer also had a slightly higher percentage of private insurance, a lower percentage of never being pregnant, a higher percentage of a family history of breast cancer, and a higher percentage of being premenopausal or being postmenopausal and having used HRT.

**Table:** Characteristics and Associations of Breast Cancer Risk With Nativity and Percentage of Life Lived in the United States Among a Sample (N = 570) of Asian American Women in the San Francisco Bay Area, Asian Community Health Initiative, 2013–2014

Characteristic	No. (%)^a^		Risk of Breast Cancer, OR (95% CI)	
	Breast Cancer Cases (n = 132)	Controls^b^ (n = 438)	Adjusted for Age and Country of Origin^c^	Fully Adjusted^d^
**Nativity and percentage of life lived in United States^e^**				
US-born	22 (17)	144 (33)	1 [Reference]	1 [Reference]
Immigrant, ≥50% of life in United States	56 (42)	110 (25)	2.94 (1.65–5.21)	3.00 (1.56–5.75)
Immigrant, <50% of life in United States	54 (41)	184 (42)	1.87 (1.03–3.37)	2.46 (1.21–4.99)
**Education**				
College graduate	81 (61)	275 (63)	1 [Reference]	1 [Reference]
Some college	21 (16)	87 (20)	0.71 (0.41–1.23)	1.10 (0.59–2.06)
High school diploma or less	30 (23)	76 (17)	1.01 (0.60–1.71)	2.27 (1.12–4.58)
**Home ownership^e^**				
Renter or non-homeowner	30 (23)	162 (37)	1 [Reference]	1 [Reference]
Homeowner	102 (77)	276 (63)	2.37 (1.48–3.81)	2.21 (1.23–3.97)
**Health insurance**				
Private insurance	102 (77)	310 (71)	1 [Reference]	1 [Reference]
Public insurance or not insured	30 (23)	128 (29)	0.47 (0.28–0.77)	0.50 (0.26–0.98)
**Pregnancy history^e^**				
Age at first birth <25 y	18 (14)	81 (18)	1 [Reference]	1 [Reference]
Age at first birth 25–29 y	46 (35)	101 (23)	2.34 (1.23–4.48)	1.90 (0.93–3.85)
Age at first birth 30–34 y	24 (18)	95 (22)	1.54 (0.74–3.18)	0.91 (0.41–2.04)
Age at first birth ≥35 y	21 (16)	41 (9)	3.21 (1.46–7.05)	3.14 (1.29–7.63)
Never had a pregnancy that lasted ≥7 months	23 (17)	120 (27)	1.24 (0.60–2.56)	1.15 (0.51–2.59)
**Any family history of breast cancer^e^^,^ f**				
No	100 (76)	384 (88)	1 [Reference]	1 [Reference]
Yes	32 (24)	54 (12)	2.05 (1.24–3.38)	2.45 (1.38–4.36)
**Menopausal status and use of HRT**				
Postmenopausal, no HRT	41 (31)	196 (45)	1 [Reference]	1 [Reference]
Premenopausal	71 (54)	213 (49)	9.27 (3.86–22.2)	10.9 (4.40–26.9)
Postmenopausal, used HRT	20 (15)	29 (7)	2.15 (1.06–4.36)	2.04 (0.93–4.48)
**Body mass index, kg/m^2^**				
<23	61 (46)	221 (50)	1 [Reference]	1 [Reference]
23–26	53 (40)	143 (33)	1.29 (0.83–1.98)	1.47 (0.90–2.41)
≥27	18 (14)	74 (17)	0.84 (0.46–1.55)	1.02 (0.52–2.02)

Abbreviation: HRT, hormone replacement therapy.

a Percentages may not add to 100% because of rounding.

b Asian American women in the San Francisco Bay Area without a diagnosis of breast cancer.

c Each variable entered individually into a logistic regression model adjusted for age group (20–39, 40–59, or ≥60 years) and country of origin (Chinese, Filipina, or other Asian).

d Logistic regression model adjusted for age group, country of origin, and all variables in the table.

e Number of breast cancer cases and controls differed significantly on this variable on the basis of χ^2^ test (*P* < .05).

f Family history was defined as a mother, sister, or daughter with breast cancer.

Minimally adjusted ORs show that immigrant Asian American women had higher risk of breast cancer than US-born Asian American women (OR = 2.94 [95% CI, 1.65–5.21] for immigrant Asian American women who lived ≥50% of their life in the United States; OR = 1.87 [95% CI, 1.03–3.37] for immigrant Asian American women who lived <50% of their life in the United States). The risk of breast cancer was only slightly higher among immigrant Asian American women who lived ≥50% of their life in the United States compared with immigrant Asian American women who lived <50% of their life in the United States (OR = 1.57 [95% CI, 0.99–2.50], *P* = .06), but the lower limit of the CI just included the null. After we adjusted for potential confounders in the fully adjusted model, immigrant Asian American women still had higher risk of breast cancer than US-born Asian American women (OR = 3.00 [95% CI, 1.56–5.75] for immigrant Asian American women who lived ≥50% of their life in the United States; OR = 2.46 [95% CI, 1.21–4.99] for immigrant Asian American women who lived <50% of their life in the United States). We found no difference in fully adjusted odds ratios of having breast cancer between the 2 immigrant groups (OR = 1.22 [95% CI, 0.70–2.15]).

In the fully adjusted model, home ownership and having a high school diploma or less were associated with greater breast cancer risk. The association between education and breast cancer risk was significant only after we adjusted for menopausal status, because premenopausal women had higher levels of education than postmenopausal women. Excluding either menopausal status or education did not change the main estimated effect between breast cancer and nativity with percentage of life lived in the United States, so we included both variables in the final model. Women who gave birth to their first child when they were 35 or older had higher breast cancer risk than those who gave birth to their first child when they were younger than 25. Having an immediate family member with breast cancer was associated with higher odds of having breast cancer. Premenopausal women had higher risk of breast cancer than postmenopausal women who had never used HRT.

## Discussion

This study provides preliminary evidence that immigrant Asian American women have a higher risk of breast cancer than US-born Asian American women. This finding confirms our first hypothesis that breast cancer risk among Asian American women would differ by nativity. However, this finding is contrary to earlier studies of Asian American populations in California showing that immigrants had lower rates of breast cancer than US-born women (1,2).

Our findings did not fully support the second hypothesis, that greater percentage of life lived in the United States would be associated with greater breast cancer risk among women who were immigrants, as suggested by prior research (1,20,21). The result for the logistic regression model that controlled only for age and Asian country of origin showed that greater percentage of life lived in the United States was not significantly associated with greater breast cancer risk, although the direction of the odds ratio suggested that there might be a slight association. Nevertheless, percentage of life lived in the United States was not associated with greater breast cancer risk after controlling for other variables in the model.

The third hypothesis was that modifiable risk factors for breast cancer — including pregnancy history, use of HRT, and BMI — would attenuate the differences in breast cancer risk by nativity and percentage of life lived in the United States. The association between immigrant status and greater breast cancer risk remained, even after adjusting for these known breast cancer risk factors. Modifiable risk factors are often cited as possible reasons for increased breast cancer risk that occurs with greater acculturation (1,3,7). Our findings indicate that yet-unidentified risk factors may exist among Asian American immigrants, leading to higher breast cancer risk than among their US-born counterparts.

Questions remain about why immigrant Asian American women had higher risk of breast cancer than US-born Asian American women in our sample. Data from CHIS 2012 in the same geographic area found that among Asian Americans, immigrant women were more likely than US-born women to have had a mammogram in the previous 2 years (63.2% vs 37.9%) (22), suggesting that our findings may be in part due to higher rates of detection among immigrant women.

Secular changes in breast cancer risk factors and the resulting increases in breast cancer rates in Asian countries, especially in affluent areas, may further explain our finding (3,4). The current Asian American immigrant population in the San Francisco Bay Area may reflect trends among populations in Asia. CHIS data show that the percentage of naturalized Asian Americans in the San Francisco Bay Area with household incomes greater than $135,000 increased from 18% in 2005 to 42% in 2016 (23). Several studies have found higher socioeconomic status, measured by income and education, to be associated with greater risk for breast cancer (10,24). Therefore, recent Asian immigrants to the San Francisco Bay Area may be arriving in the United States with higher risk for breast cancer than was found previously.

Other findings in our study coincide with previous findings on breast cancer among Asian American women. Greater breast cancer risk was associated with higher socioeconomic status, measured as home ownership and health insurance status (10,24). Giving birth to one’s first child when aged 35 or older, compared with giving birth for the first time when younger than 25, was associated with higher risk for breast cancer (7,25). Having a family history of breast cancer was associated with higher risk of breast cancer (26). Premenopausal women, compared with postmenopausal women who had never used HRT, had higher risk of breast cancer, which reflects research showing higher rates of breast cancer among premenopausal Asian American women (1).

Our study had several limitations. One limitation was the relatively small sample size. A second was that the case and control subsamples were matched according to Asian country of origin, so we were unable to disaggregate the various Asian American subpopulations. A third limitation was the case-control design, which did not allow us to examine trends over time. Lastly, our study was conducted only in the San Francisco Bay Area, so our results may not be generalizable to the larger population of Asian American women in the United States. The San Francisco Bay Area is unique in its relative affluence and the characteristics of the Asian immigrants it attracts, so our findings may not apply to other areas of the United States, especially less affluent areas. Nevertheless, as with the research on breast cancer in Marin County in the San Francisco Bay Area (27,28), the unique demographics of the region can point to important disease associations and lead to the discovery of new risk factors. A major strength of this study was the use of a population-based survey conducted in multiple languages that was designed to examine breast cancer risk among Asian American women in the San Francisco Bay Area.

Clinicians who serve Asian American patients should be aware of the potential trend of higher breast cancer risk among immigrant Asian American women and allocate resources for breast cancer treatment among this demographic accordingly. Interventions are needed to increase breast cancer screening among both immigrant and US-born Asian American women to prevent breast cancer from progressing. Immigrant Asian American women likely have different barriers to screening and treatment than US-born Asian American women, including language and culturally appropriate care.

Future studies are needed to corroborate the novel findings of our research. Studies using larger samples in broader geographic areas should compare breast cancer risk among Asian Americans by nativity and explore possible explanations for differences. Cross-national studies that examine breast cancer risk in the country of origin and upon immigrating to the United States would be useful. Such research could illuminate our understanding of how breast cancer risk changes over time, especially in an environment of international migration and changing contextual risk factors. Such investigations can lead to better breast cancer prevention activities, especially among immigrant groups living in the United States.
